# Supplementary material for: Pigmented Microbial Extract (PMB) from Exiguobacterium Species MB2 Strain (PMB1) and Bacillus subtilis Strain MB1 (PMB2) Inhibited Breast Cancer Cells Growth In Vivo and In Vitro
Source: Int J Mol Sci. 2023 Dec 12;24(24):17412. doi: 10.3390/ijms242417412 (PMC10743659; doi:10.3390/ijms242417412)
Supplement: Supplementary file 1 [file ijms-24-17412-s001.zip › ijms-2700462-supplementary.pdf]

Supplementary information

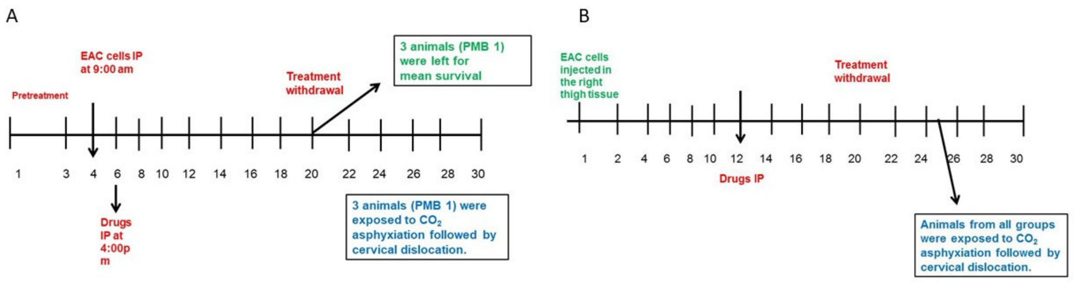

Figure S1A. Study design of EAC liquid tumor model in Swiss albino mice. **S1B:** Study design of EAC solid tumor model in Swiss albino mice.

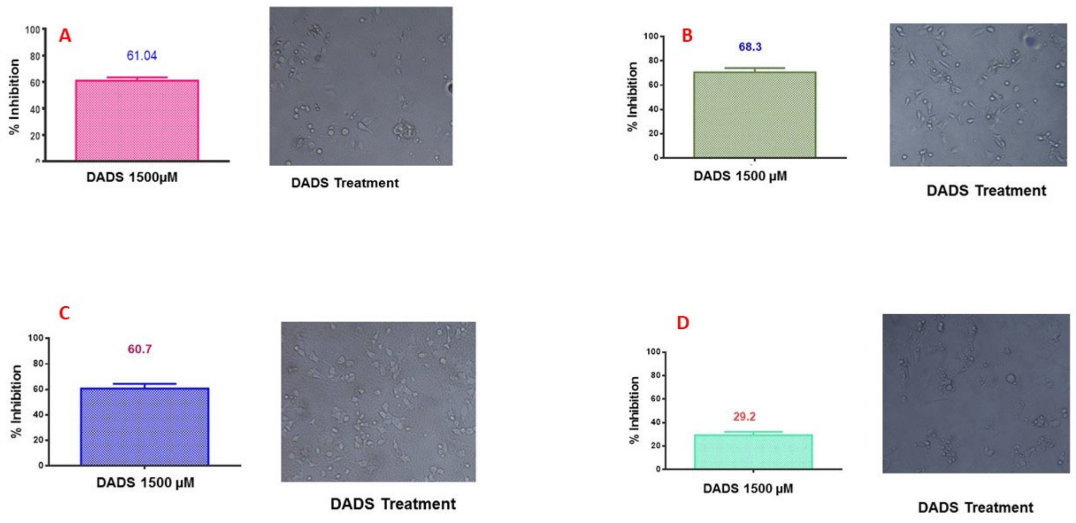

Figure S2. DADS (1500μM) decreased the viability of MDA-MB-468 (S2A), MDA-MB-231 (S2B), and MCF-7 (S2C) cells. DADS has also reduced the viability of normal cell line BEAS-2B (S2D).

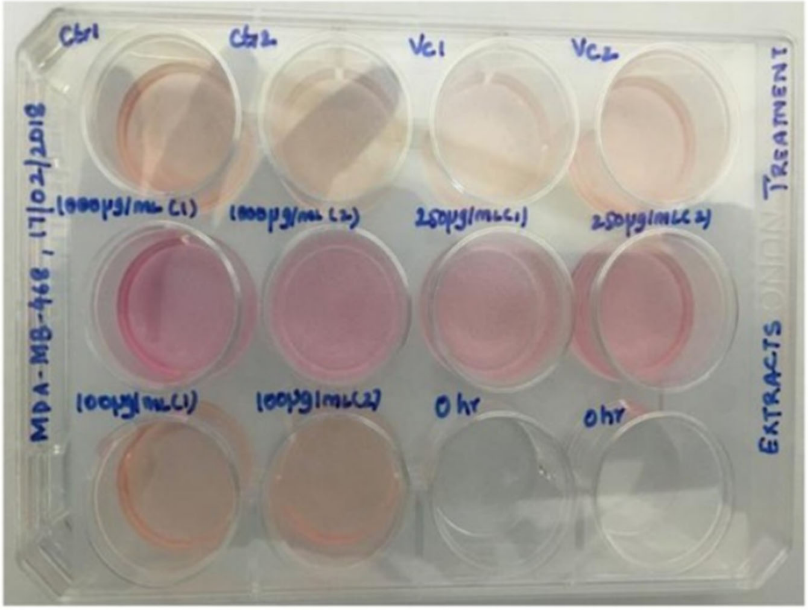

Figure S3. Cytostatic effect of the microbial extract on MDA MB-468 cell growth. MDA-MB-468 cells were treated with the increasing concentration of the microbial extract (100 µg/ml, 250 µg/ml and 1000µg/ml) for 48 hours in six well plates. The media color in the treatment group showed less consumption of nutrients as evidenced by pink color compared to control /VC wherein the medium is pale yellow in color.

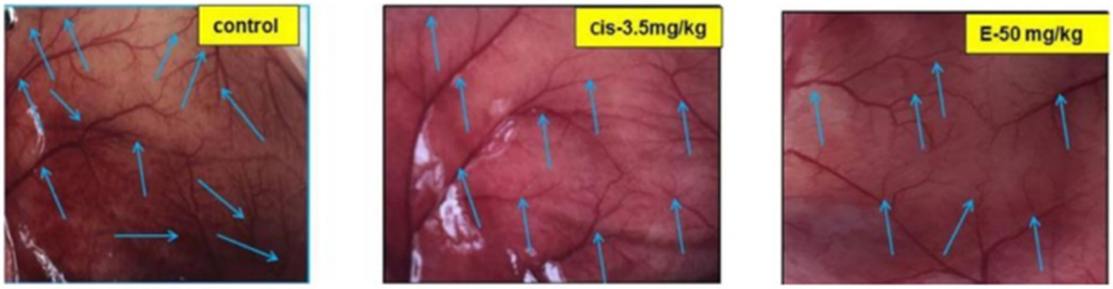

Figure S4. **PMB1 reduced angiogenesis in mice**. The effect of microbial extract on angiogenesis was examined in the peritoneum of EAC-xenografted mice. PMB1 inhibited the formation of blood vessels at the concentration of 50 mg/kg body weight.

List of Supplementary Tables:

Table S1. EAC liquid tumor model study groups. Number of mice in each group = 5.

**Group**	**Treatment**
Normal	Untreated
Control	EAC
Standard (Positive Control)	EAC + Cisplatin 3.5 mg/kg
PMB1(High Dose)	EAC + PMB1 100mg/kg
PMB1(Low Dose)	EAC + PMB1 50mg/kg

Table S2. EAC solid tumor model study groups. Number of mice in each group = 5.

**Group**	**Treatment**
Normal	Untreated
Control	EAC
Standard (Positive Control)	EAC + Cisplatin 3.5 mg/kg
PMB1(High Dose)	EAC + PMB1 50mg/kg
PMB1(Low Dose)	EAC + PMB1 25mg/kg

Table S3. IC-50 values of microbial extracts PMB1 and PMB2 against BC cell lines and normal lung epithelial cell line BEAS-2B.

**IC-50 values of pharmacological agents tested**				
**Inhibitor**	**MDA-MB-468**	**MDA-MB-231**	**MCF-7**	**BEAS-2B**
PMB1(µg/mL)	795	939.3	>2000	>2000
PMB2(µg/mL)	>2000	>2000	>2000	>2000

Table S4. Average body weight of Swiss albino mice on the day of sacrifice.

**Treatment**	**Dose**	**Average body weight on day 20**
**Normal**	Untreated	22.6 ± 0.48
**Control**	EAC untreated	45.1 ± 2.46
**Cisplatin**	3.5 mg/kg	33.1 ± 0.64
**PMB1**	50 mg/kg	28.2 ± 1.24
